# Comprehensive Genome-Wide Identification and Expression Profiling of *bHLH* Transcription Factors in *Areca catechu* Under Abiotic Stress

**DOI:** 10.3390/ijms252312936

**Published:** 2024-12-01

**Authors:** Akhtar Ali, Noor Muhammad Khan, Yiqi Jiang, Guangzhen Zhou, Yinglang Wan

**Affiliations:** 1 Hainan Key Laboratory for Sustainable Utilization of Tropical Bioresources, School of Tropical Agriculture and Forestry, Hainan University, Haikou 570228, China; anaakhtar631@gmail.com (A.A.); noormaghmoom@gmail.com (N.M.K.); jyq217317@163.com (Y.J.); 2 The Ministry of Education Key Laboratory for Ecology of Tropical Islands, Key Laboratory of Tropical Animal and Plant Ecology of Hainan Province, College of Life Sciences, Hainan Normal University, Haikou 571158, China; gzzhou@hainanu.edu.cn

**Keywords:** *Areca catechu*, *bHLH* gene family, genome-wide analysis, expression pattern, abiotic stresses

## Abstract

The basic helix-loop-helix (*bHLH*) transcription factor (TF) family, the second-largest among eukaryotes, is known for its evolutionary and functional diversity across plant species. However, *bHLH* genes have not yet been characterized in *Areca catechu*. In this study, we identified 76 *AcbHLH* genes, which exhibit a variety of physicochemical properties. Phylogenetic analysis revealed evolutionary relationships between *Arabidopsis thaliana bHLH* genes (*AtbHLH*) and their counterparts in *A. catechu* (*AcbHLH*). These analyses also highlighted conserved amino acid motifs (S, R, K, P, L, A, G, and D), conserved domains, and evolutionary changes, such as insertions, deletions, and exon gains or losses. Promoter analysis of *AcbHLH* genes revealed 76 cis-elements related to growth, phytohormones, light, and stress. Gene duplication analysis revealed four tandem duplications and twenty-three segmental duplications, while *AcbHLH63* in the Areca genome exhibited significant synteny with *bHLH* genes from *A. thaliana*, *Vitis vinifera*, *Solanum lycopersicum*, *Brachypodium distachyon*, *Oryza sativa*, and *Zea mays*. Furthermore, relative expression analysis showed that under drought stress (DS), *AcbHLH22*, *AcbHLH39*, *AcbHLH45*, and *AcbHLH58* showed distinct upregulation in leaves at specific time points, while all nine *AcbHLH* genes were upregulated in roots. Under salt stress (SS), *AcbHLH22*, *AcbHLH39*, *AcbHLH45*, and *AcbHLH58* were upregulated in leaves, and *AcbHLH22*, *AcbHLH34*, and *AcbHLH39* exhibited differential expression in roots at various time points. This study provides valuable insights into the bHLH superfamily in *A. catechu*, offering a solid foundation for further investigation into its role in responding to abiotic stresses.

## 1. Introduction

Transcription factors (TFs) are pivotal in regulating plant growth and development by controlling gene expression [1,2]. They influence various cellular processes by interacting with other proteins involved in transcription [3]. The bHLH is the second-most abundant group of TFs family found in plants, animals, and microorganisms within this group of proteins [4]. The bHLH transcription factors domain consists of 50–60 amino acids in length and is divided into two functional regions, the N-terminal basic domain and the C-terminal (HLH) domain region [5]. The N-terminal basic region consists of 10–15 bp amino acids and is responsible for binding to cis-elements E-box (CANNTC), while the C-terminal region consists of 40–50 bp amino acids and is responsible for the formation of homo- and heterodimer protein complexes [6]. Moreover, MYC-like bHLH proteins possess an MYB-interacting region and extra N-terminal MYC domain, enabling attachment to bHLH and R2R3-MYB domain proteins, respectively [7].

Many physiological and biochemical processes in plants have been linked to bHLH TFs, as shown by the extensive study on these proteins [8]. Key developmental processes such as seed coat differentiation [9], stomata differentiation [10,11], trichome/root hair creation [12], fruit development [13,14], and carpel edge development are regulated by transcription. Certain bHLH TFs also have a role in premature seedling photomorphogenesis [15,16]. Additionally, a growing body of research indicates the involvement of bHLH proteins in plant actions connected to the heterogeneity of abiotic stressors, for instance, salinity, drought, low temperature, and mechanical injury [14,17]. In addition, bHLH proteins have been extensively studied in plants for their regulatory functions in forming secondary metabolites, including alkaloids [18], flavonoids [19], phenolic acids [20], and terpenoids [21]. The advancement in sequencing technologies facilitated the recognition and characterization of numerous families (bHLH) in plant genomes. Genome-wide analyses of the bHLH family have been identified in various plants, including *Arabidopsis* [22] *O. sativa* [23] *S. lycopersicum* [24], *Z. mays* [25], *T. aestivum* [26], *P. alba* [27], *S. tuberosum* [28], and other plants based on genome sequences [25].

*A. catechu,* is a vital evergreen vascular monocot tree belonging to the Areceae family [29]. *A. catechu* possesses significant medicinal and economic importance and is extensively cultivated in China, India, Thailand, Indonesia, Malaysia, and Cambodia [30,31]. In China, the areca nut is utilized in traditional Chinese medicine [32]. Due to its high arecoline, the areca nut has become a significant cash crop in Southeast Asia and Africa as the fourth most addictive substance in the world after alcohol, caffeine, and nicotine. Additionally, the study of bHLH protein *A. catechu*, a rare monocot tree, not only carries substantial economic value, but also holds theoretical importance in deciphering abiotic stress responses within this plant species [33]. However, a comprehensive study of *AcbHLH* gene expression patterns within the *Areca* genome remains an understudied area within the scientific literature. This research categorized 76 *AcbHLHs* genes from the *A. catechu* genome. The comprehensive analyses were conducted on their phylogenic relationships, sequence characteristics, gene structures, promoter sequences, and collinearity. Furthermore, we investigated the specific expression of these genes across various parts in response to drought and salt stresses. This work provides a base for future research on molecular mechanisms of *AcbHLH* genes in response to abiotic stresses in *A. catechu*.

## 2. Results

### 2.1. Identification of bHLH Genes in A. catechu

The present study identified and characterized 76 *AcbHLH* genes in the *A. catechu* genome. The gene names were assigned from *AcbHLH01* to *AcbHLH76* based on phylogenic connection with already known *A. thaliana bHLH* genes (162) (Table S1A). Additionally, characterizing these genes provided information on their molecular weight, isoelectric points, protein length, domain composition, and subcellular localization (Table S1B). Among 76 *AcbHLH* genes, the amino acid lengths ranged from 149 amino acids (*AcbHLH17*) up to 685 amino acids (*AcbHLH59*), while the moderate range of amino acids was 363.08 kDa. The molecular mass of the proteins ranged from 16.28 kDa (*AcbHLH17*) to 76.32 kDa (*AcbHLH59*), whereas their molecular mass ranged from 16.28 kDa (*AcbHLH17*) to 76.32 kDa (*AcbHLH59*). The calculated isoelectric point (pI) of *AcbHLH25* started from 4.99 to 10.30 (*AcbHLH57*), with a mean of 6.96 pI of *AcbHLH* genes. The subcellular localization prediction result revealed that 70 *AcbHLHs* are present in the nucleus, cytoplasm 01, chloroplast 04, and mitochondria 01 (Table S1B).

### 2.2. Phylogenetic and Multiple Sequence Alignment

**Figure 1:**
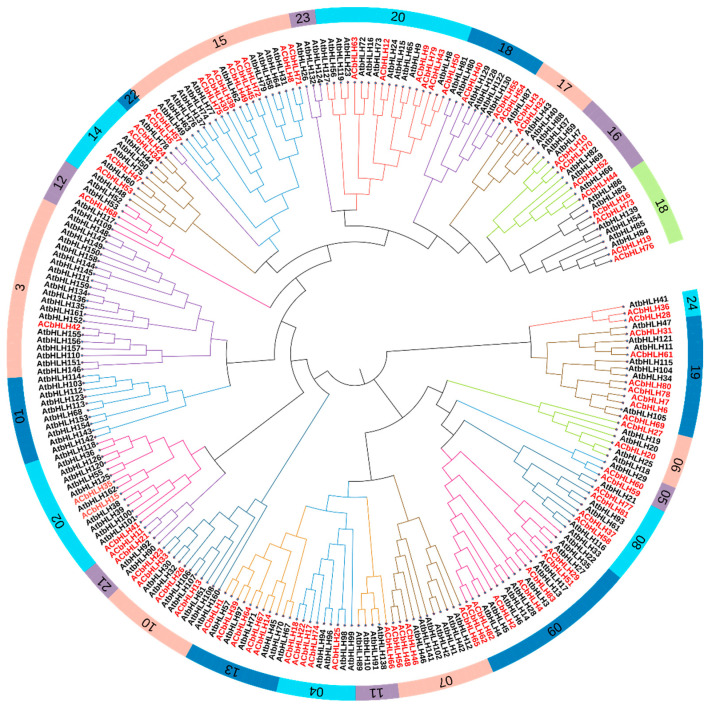
A phylogenic tree illustrates the relationship among *bHLH* domains of *A. catechu* and *A. thaliana*. The black color presents (*AtbHLH*) *A. thaliana*, and red represents *AcbHLH* of *A. catechu bHLH* protein.

The phylogenetic relationships among 76 *AcbHLH* and 162 *AtbHLH* genes were inferred using the Neighbor Joining (NJ) method with 1000 bootstrap replicates. The analysis was based on amino acid sequence data (Figure 1 and Table S1B). *bHLH* genes were classified into 24 groups based on topological structures. According to Pires and Gabriela’s presented classification method and topological outline of the tree, the phylogenetic tree of 238 *bHLH* genes was classified into 24 groups (1–24) [34]. The phylogenic tree shows a closed relationship between the *AcbHLHs* and *AtbHLHs* proteins with a bootstrap support of 80 or higher. The study reveals that in Group 19, three pairs of genes showed close evolutionary ties: *AtbHLH47* with *AcbHLH31*, *AtbHLH11* with *AcbHLH61*, and *AtbHLH105* with *AcbHLH69*. Group 8 displayed a close relationship between *AtbHLH21* and *AcbHLH77*, while Group 9 linked *AtbHLH13* with *AcbHLH83*. In Group 4, *AtbHLH98* showed a close relationship with *AcbHLH25*. Group 13 connected *AtbHLH71* with *AcbHLH64*, and Group 2 included *AtbHLH162* with *AcbHLH35*. Group 3 revealed a relationship between *AtbHLH155* and *AcbHLH42*, and Group 14 linked *AtbHLH60* with *AcbHLH47*. In Group 22, *AtbHLH78* was closely related to *AcbHLH5*, while Group 15 included two pairs: *AtbHLH77* with *AcbHLH75* and *AtbHLH62* with *AcbHLH49*. Group 20 also presented two connections, with *AtbHLH24* linked to *AcbHLH12* and *AtbHLH8* to *AcbHLH50*. Additionally, Group 17 showed an evolutionary relationship between *AtbHLH87* and *AcbHLH3*, while Group 16 connected *AtbHLH66* with *AcbHLH44*. These findings underscore the evolutionary connections between *bHLH* genes in *Arabidopsis* and their counterparts, suggesting functional and ancestral relationships across these groups.

**Figure 2:**
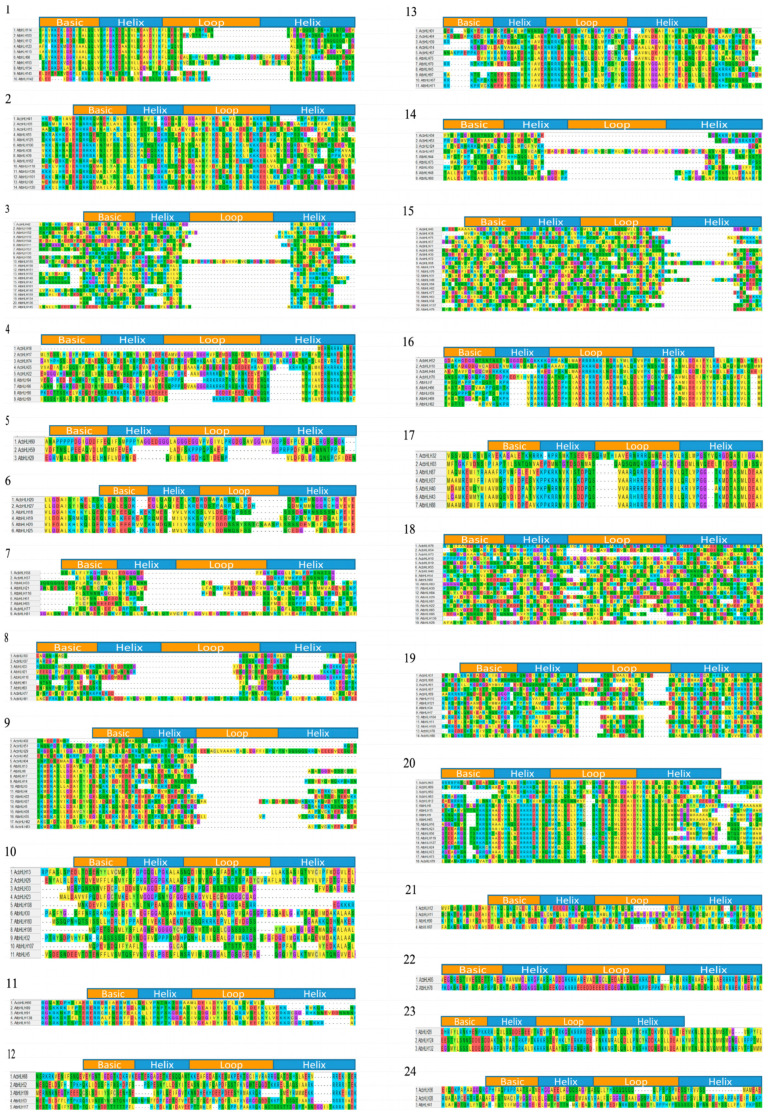
Multiple sequence alignments of *A. catechu* and 24 subgroups *A. thaliana. A. catechu* is devoid of subgroup 24. The location and boundaries of the bHLH domain are indicated at the top of each subgroup.

As illustrated in Figure 2, our sequence alignment analysis of *AcbHLH* genes in *A. catechu* revealed consistent patterns of amino acid motifs (S, R, K, P, L, A, G, E, Y, I, V, H, M, Q, N, and D). As previously observed, these motifs are shared by bHLH proteins from various plant species.

### 2.3. Analysis of Gene Structure and Conserved Domains of AcbHLH Genes

**Figure 3:**
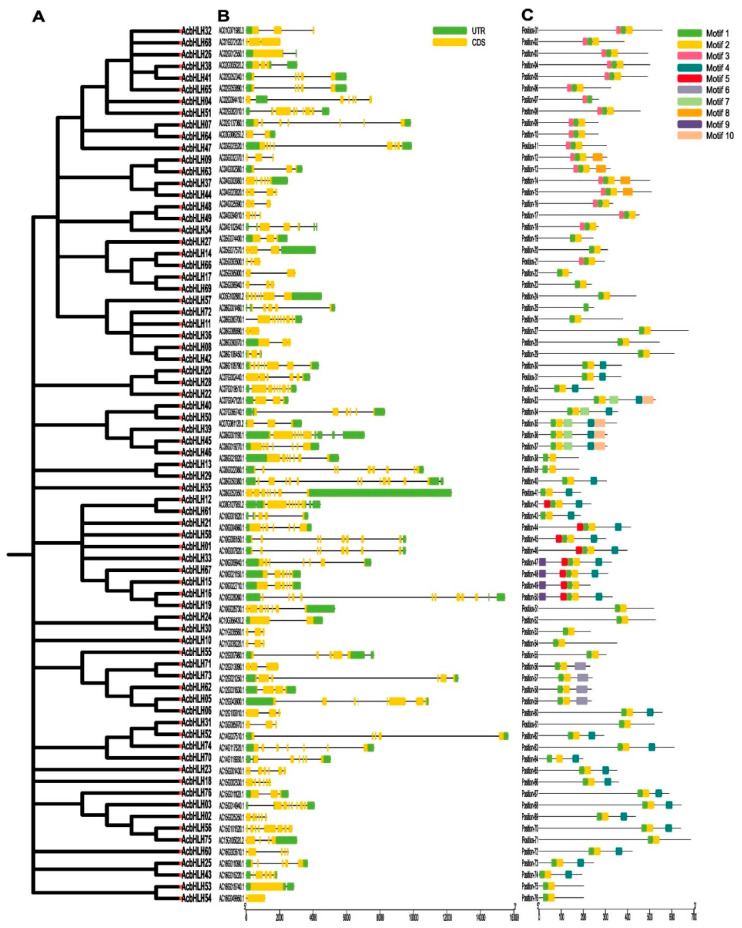
Comparative analysis of *A. catechu AcbHLH* gene phylogeny, structure, and motifs. (**A**) Phylogeny was inferred based on the NJ method with 1000 bootstrap replicates. (**B**) Introns and exons are visually represented as yellow and black lines. (**C**) Amino acid motifs (1–10) are indicated by colored, relative protein lengths represented with black lines.

To reveal the *AcbHLH* genes’ phylogeny and intron–exon structures, we compared 76 *AcbHLH* genes, ranging from 1 to 13, showing differences in exon–intron structures (Figure 3A,B and Table S1B). Of the 76 *AcbHLH* genes studied, 2 genes (2.63%) possess a single exon, while the rest exhibit two or more exons in their structures. Some intron-free genes were identified within Subgroups 2 and 6. Among the remaining 69 genes, three intron patterns were most prevalent. *AcbHLH40* exhibited the highest number of introns (13), while genes in Group 3 contained either 0 introns or a single intron. Group 3 exhibited the most significant variant in the number of exons, ranging from a single intron in *AcbHLH22* to 18 introns in *AcbHLH40*. As revealed by comparative analysis, Subgroup 3, among the analyzed *AcbHLH* genes, exhibited the most diverse range of intron counts.

Distribution analysis of motifs among *AcbHLH* gene, as shown in (Figure 3C), revealed that motifs 1, 2, and 3 were prevalent and abundant in all groups, while motifs 6, 8, 9, and 10 exhibited the least across the *AcbHLH* proteins. Distinct motif compositions characterized *AcbHLH* subgroups. For instance, Motifs 1, 2, and 3 were commonly found in Subgroup 1 and 2 proteins. Subgroup 4 proteins, on the other hand, were characterized by the presence of motifs 1, 2, 5, and 9. Some motifs were notably prevalent within particular subgroups, such as motifs 8, 10, and 6 in subgroups 2, 3, and 7, respectively. Motifs 1 and 2 mainly were observed across multiple subgroups. Additional analysis identified distinct patterns in the arrangement and relative positions of the motifs. For instance, motif 4 exclusively appears at the end of subgroups 3, 5, and 6, while a specific arrangement has been observed in motifs 1, 2, and 10.

### 2.4. Characterization of Cis-Acting Elements Within AcbHLH Promoter Regions

**Figure 4:**
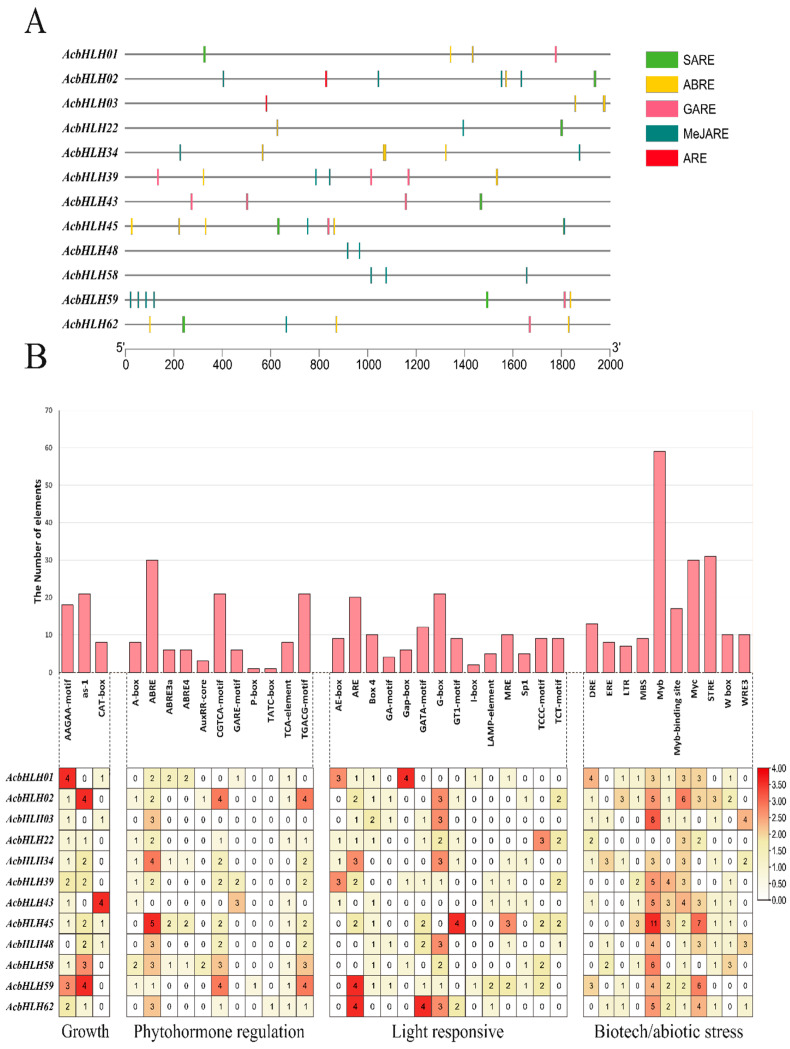
(**A**) Cis-regulatory elements in the 2000 bp upstream region of *AcbHLH* gene promoters. (**B**) Distribution of cis—regulatory elements of *AcbHLH* gene members. Colored rectangles visually depict the various cis-acting elements.

To investigate the functional activities and regulatory regions of *AcbHLH* genes, we analyzed the 2000 bp upstream promoter regions for potential cis-elements using PlantCARE (Figure 4). Our analysis identified significant stress-related cis-elements within these regions, suggesting a critical role for *AcbHLH* genes in stress response pathways. We categorized the cis-acting elements into four functional groups as depicted in Figure 4B: (I) growth-related, (II) phytohormone-responsive, (III) light-responsive, and (IV) stress-responsive elements. The growth-related group includes motifs such as AAGAA-motif, as-1, and CAT-box. The phytohormone-responsive group contains elements associated with various hormones, such as ABRE, ABRE3a, ABRE4, AuxRR-core, and GARE-motif. Light-responsive elements include AE-box, ARE, Box 4, GA-motif, Gap-box, GATA-motif, G-box, GT1-motif, I-box, Lamp-element, MRE, Sp1, TCCC-motif, and TCT-motif. The stress-responsive category includes DRE, ERE, LTR, MBS, Myb, Myb-binding sites, Myc, STRE, W box, and WRE3.

This analysis highlights the potential involvement of *AcbHLH* genes in regulating responses to environmental stimuli. Notably, cis-elements associated with abscisic acid (ABA) and salicylic acid (SA) were identified within the promoter regions of *AcbHLH* genes linked to abiotic stress responses, such as extreme temperatures, wounding, and drought. These hormone-responsive elements further suggest involvement in pathways regulated by SA, gibberellin, methyl jasmonate (MeJA), auxin, and ethylene (Figure 4A).

### 2.5. Chromosomal Distribution and Gene Duplication of AcbHLH Genes

**Figure 5:**
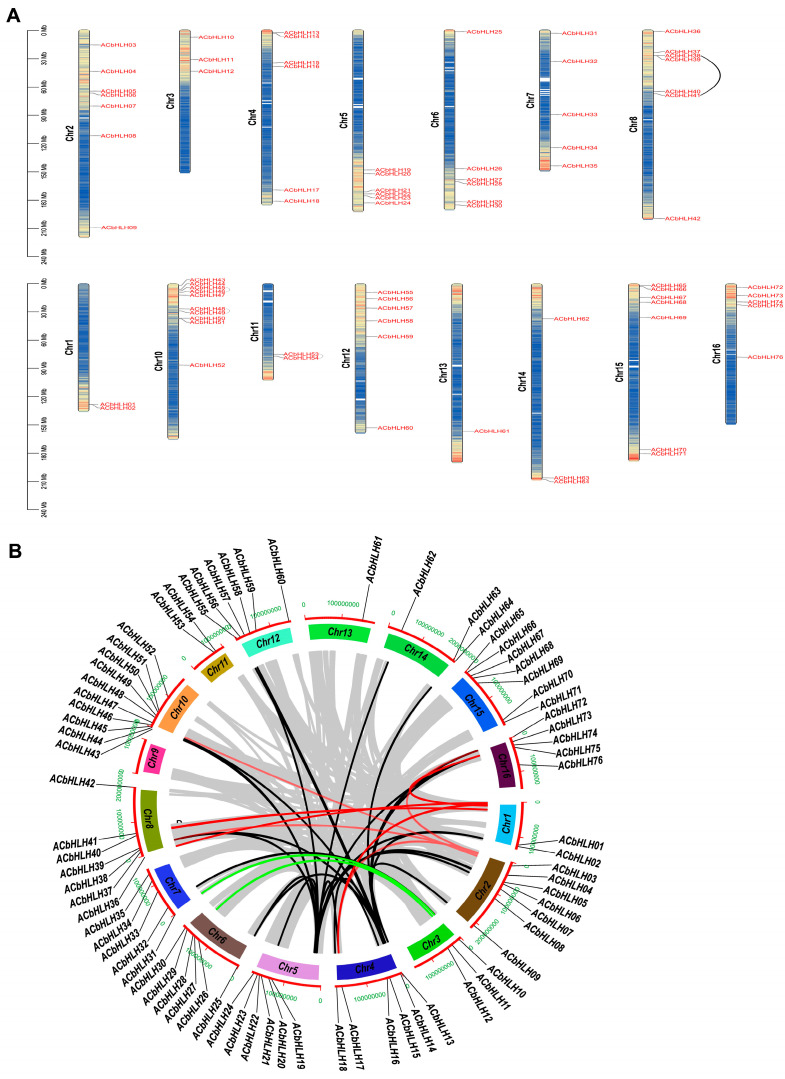
(**A**) Location of 76 *AcbHLH* genes across 16 *A. catechu* chromosomes. The left-hand scale indicates chromosomal length. (**B**) The schematic diagram represents the distribution of *A. catechu* chromosomes and interchromosomal interaction. Distinct colored lines within the diagram represent gene pairs. Red lines indicate *AcbHLH* gene pairs. *A. catechu* are labeled outside the chromosome circles, while chromosome numbers are indicated within.

The latest *A. catechu* genome database enabled the creation of a precise physical map that offers the specific positions of *AcbHLH* genes within the genome (Figure 5A and Table S2). The allocation of 76 *AcbHLH* genes across Chr1 to 16 displayed uneven distribution patterns. The name of each *AcbHLH* gene was assigned based on its sequential physical location along *A. Catechu* Chr1-10, from top to bottom. Chr10 possesses the greatest number of *AcbHLH* genes (10 genes, ~13.16%), followed by Chr 2, 8, and 16, each containing 7, respectively (~9.21%), while Chr13 contained the least (1, ~3.31%) and Chr9 contain zero genes. Chr4, Chr5, Chr6, and Chr12 each contained 6 (~7.9%) *AcbHLH* genes. Chr7 and Chr15 each contained 5 (~6.6%) *AcbHLH* genes. Chr3 and Chr14 contained 3 (~3.94%) and chr11 (2~2.6%) *AcbHLH* genes. In addition, we discussed the chr of *AcbHLH* gene-duplication instances. The chromosomal region spanning 30 Mb and 60 Mb exhibiting two homogenous genomic segments is clarified as a tandem duplication event [35]. On Chr8 and 11, we observed 04 tandem duplication segments involving two genes *AcbHLH* (Figure 5A). *AcbHLH38* and *AcbHLH41* on Chr 8, and *AcbHLH53* and *AcbHLH54* on chromosome 11, each had tandem repeat events, while Chr10 had two tandem duplication events involving four *AcbHLH* genes (*AcbHLH45*, *AcbHLH46*, *AcbHLH48* and *AcbHLH49*). The gene involved in the tandem repeat occurrences originated exclusively within identical subfamilies. For instance, genes *AcbHLH38* and *AcHLH41* had tandem repeat sequences and clustered together in subfamilies 15 and 2, *AcbHLH53* and *AcbHLH54* were clustered together in subfamily 14 and 18, while *AcbHLH45*, *AcbHLH46*, *AcbHLH48,* and *AcbHLH49* were clustered together in subfamily 15, 7, 7, and 15, respectively (Figure 5B and Table S2).

**Figure 6:**
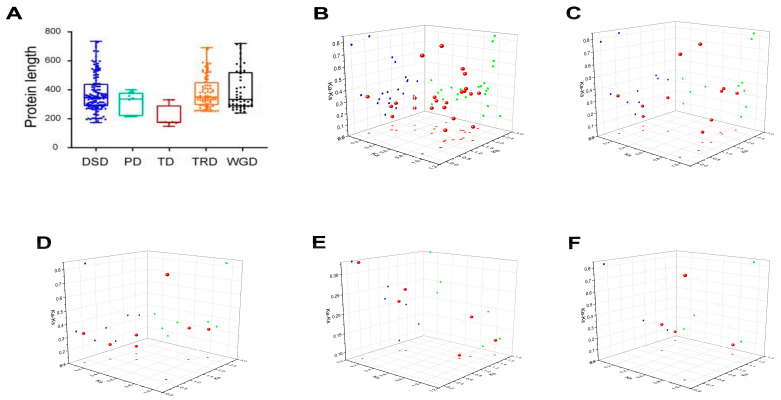
Gene *AcbHLH* duplication events. (**A**) Length distribution of *AcbHLHs* across five duplication events. (**B**–**F**) distribution of Ka, Ks, and Ka/Ks value in duplicated genes across five duplication events. (**B**–**F**) DSD, WGD, TRD, TD and PD event, respectively. Further details on the duplicated genes across the five duplication events are provided in Table S9.

Additionally, a total of 23 segmental duplications were identified within the *AcbHLH* gene family. As illustrated in Figure 6, the *AcbHLH* gene family contains 10 (13.15%) paralogs, indicating a common evolutionary origin of these *AcbHLH* members. The distribution of *AcbHLH* genes across the 16 linkage groups (LGs) of *A. catechu* was notably uneven (Figure 4). Some linkage groups, specifically LG10, d followed by LG8, exhibited a higher count of *AcbHLH* genes than others. LG2 boasted the highest count of *AcbHLH* genes, at fourteen, while LG5 contained the fewest count of one *AcbHLH*. Further analysis of *bHLH* families observed that many were significantly linked within their respective subfamilies, except *AcbHLH61*/01 and *AcbHLH062*/42. Within all identified *AcbHLH* genes, group 15 exhibited the highest count of linked genes, encompassing 9/76 genes. Additionally, group 15 has nine genes, while groups 22, 12, 11, and 3 have only one, and groups 23 and 1 have no genes (Table S2). The present study implies that genes *AcbHLH* indicate gene duplication events and have been a significant factor in the *AcbHLH* genes in *A. catechu*, resulting in the emergence of novel functions and the expansion of the *AcbHLH* gene family.

### 2.6. Analysis of Gene Duplication Events of AcbHLH Genes

To investigate the possible driving force for the diversifications of the *AcbHLH* gene family, the Dup-206 Gene finder was used to examine gene-duplication segments, inclusive of DSD, WGD, TRD, TD and PD. The result revealed that there was high variation in the number of duplicated genes and the distribution of their protein. Furthermore, the largest number of duplicated genes, at 23, is in DSD, while the lowest (4) were observed in PD. Additionally, 11 duplicated genes were identified in WGD (Figure 6 and Table S3A–C). The value of the (Ks) and (Ka) substitution rate, and the Ka/Ks ratio of the duplicated genes over five replication events was calculated, to provide insight into the selection pressure on *AcbHLH* genes. The Ka/Ks value was less than 1, indicating that clear selection of these genes has taken place, as shown in Figure 6B–F.

### 2.7. Synteny Analysis of AcbHLH Genes

**Figure 7:**
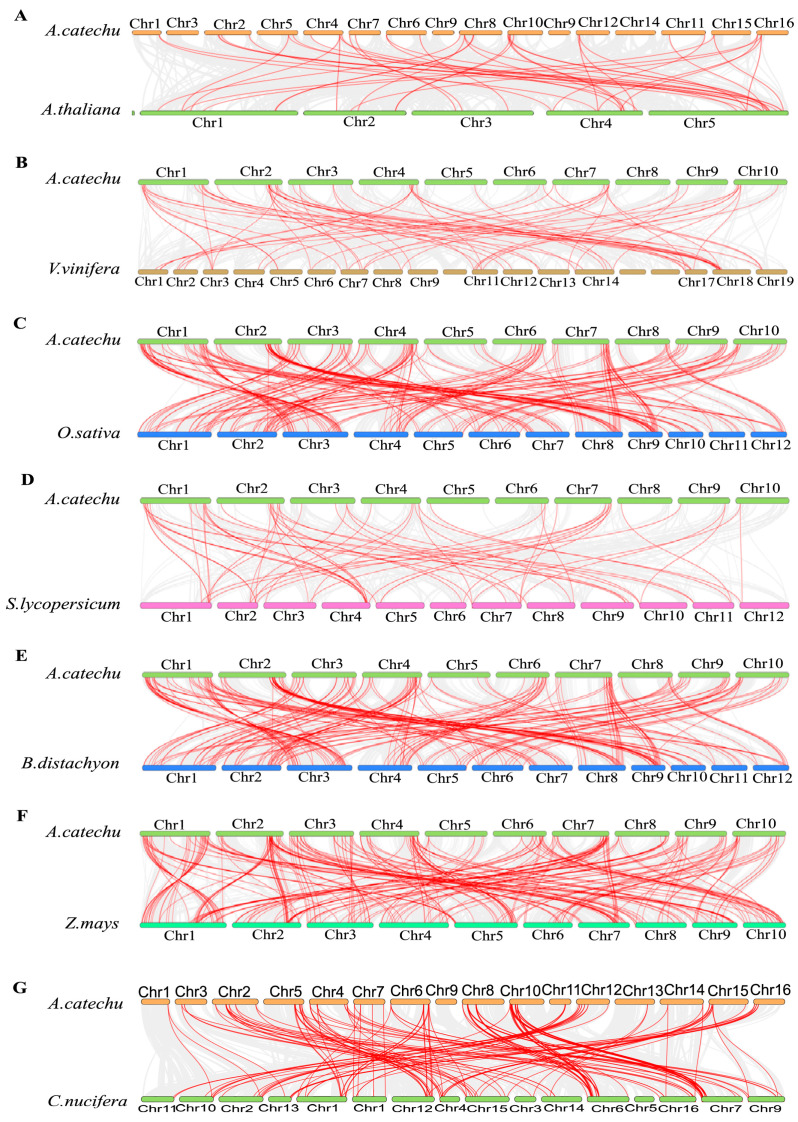
(**A**–**G**) Comparative synteny analysis of *AcbHLH* genes between *A. catechu* and six representative plant species (*A. thaliana*, *V. vinifera*, *S. lycopersicum*, *B. distachyon*, *O. sativa* sub sp. *indica*, *Z. mays* and *C. nucifera*). Gray lines show conserved syntenic blocks between *A. catechu* and other plant genomes. Red lines indicate that *AcbHLH* pairs of genes are syntenic across species.

To elucidate the phylogenic processes underlying the *A. catechu bHLH* gene family, we developed seven comparative synteny maps for *A. catechu* association, encompassing three dicots, (*A. thaliana, V. vinifera,* and *S. lycopersicum*) and four monocots (*B. distachyon, O. sativa*, *Z. mays,* and *C. nucifera*) (Figure 7 and Table S4A–F). Through collinear analysis, 63 genes of *AcbHLH* were identified, with the gene set of *A. thaliana* (41), *V. vinifera* (66), *S. lycopersicum* (59), *B. distachyon* (193), *O. sativa* (150), and *Z. mays* (170). The homologous logarithmic values for six species were shown as *A. thaliana* (44), *V. vinifera* (66), *S. lycopersicum* (60), *B. distachyon* (194), *O. sativa* (207), *Z. mays* (273) and *C. nucifera* (160). A notable finding is that several *AcbHLH* were connected with at least three synthetic gene pairs, emphasizing the link between *A. catechu* and *V. vinifera*, *AcbHLH43*, and *AcbHLH64*. *AcbHLH* gene, along with *A. thaliana* (54.0%), *B. distachyon* (53.73%), and *O. sativa* (55.56%). With regard to two or more synthetic gene pairs, the presence of these genes within the homologous gene pairs, comprising over 50% of the total, underscores their importance in evolutionary pathways.

As expected, some *AcbHLH* genes, especially *AcbHLH73*, *AcbHLH71*, *AcbHLH67*, and *AcbHLH29*, formed entirely homologous genes, with three dicotyledons. The fact that all these genes, such as *AcbHLH73*, *AcbHLH71*, *AcbHLH67*, and *AcbHLH29* exhibited the presence of homologous gene pairs with three typical dicotyledons indicated a possible evolutionary lineage leading to the development of dicotyledons. A specific *AcbHLH* gene homology with at least one gene in the five specified species was observed; for instance, *AcbHLH37*, *AcbHLH30*, *AcbHLH21*, *AcbHLH73*, *AcbHLH17*, *AcbHLH42*, *AcbHLH3*, and *AcbHLH41*. This observation suggested that these might be crucial primordial gene which have either been lost or which showed highly significant differentiation during the *A. catechu* long-term evolutionary history.

### 2.8. The Expression Patterns of AcbHLH Genes Differ Across Tissues

**Figure 8:**
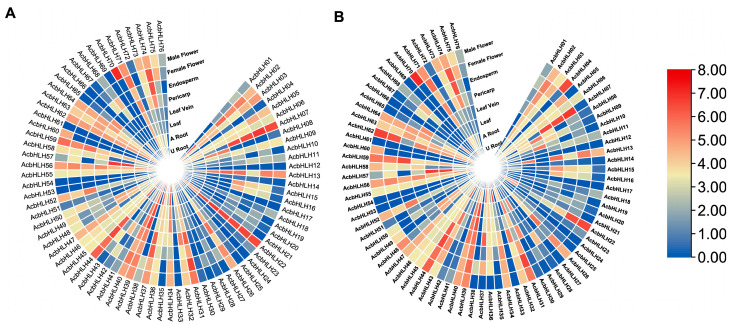
Heatmap of *AcbHLH* gene expression in *A. catechu* during salt stress (SS) (**A**) and drought stress (DS) (**B**). Red indicates larger log2FPKM values, whereas blue indicates lower values.

*A. catechu* plants were treated to drought (25% PEG6000) and salt (5% NaCl) stress, and transcriptome data were used to analyze the expression levels of *AcbHLH* genes in various tissues (Figure 8A and Table S5A,C). Under salt stress, *AcbHLH22* was the most highly expressed in all tissues, followed by *AcbHLH07*, *AcbHLH31*, and *AcbHLH44* in the pericarp, endosperm, and male and female flowers. *AcbHLH70*, *AcbHLH74*, *AcbHLH59*, *AcbHLH66*, *AcbHLH40*, *AcbHLH39*, and *AcbHLH13* were found to be highly expressed in flowers, whereas *AcbHLH74*, *AcbHLH59*, *AcbHLH52*, and *AcbHLH37* were prominent in the endosperm. *AcbHLH02*, *AcbHLH13*, *AcbHLH26*, and *AcbHLH37* were highly expressed in the pericarp, whereas *AcbHLH57*, *AcbHLH40*, *AcbHLH39*, *AcbHLH13*, and *AcbHLH08* were substantially expressed in the leaves. *AcbHLH02*, *AcbHLH34*, *AcbHLH37*, *AcbHLH44*, and *AcbHLH56* were highly expressed in roots.

*AcbHLH22*, *AcbHLH07*, *AcbHLH44*, *AcbHLH59*, and *AcbHLH62* were the most expressed genes in male and female flowers during drought stress, followed by *AcbHLH04* and *AcbHLH70*. *AcbHLH07*, *AcbHLH44*, *AcbHLH59*, and *AcbHLH74* were abundant in the endosperm. *AcbHLH07*, *AcbHLH44*, *AcbHLH13*, *AcbHLH26*, *AcbHLH37*, and *AcbHLH63* all demonstrated high expression levels in the pericarp. *AcbHLH39* and *AcbHLH57* were highly expressed in the leaf veins, followed by *AcbHLH40* (Figure 8B and Table S5B,D).

### 2.9. Phenotypic and Physiological Changes

**Figure 9:**
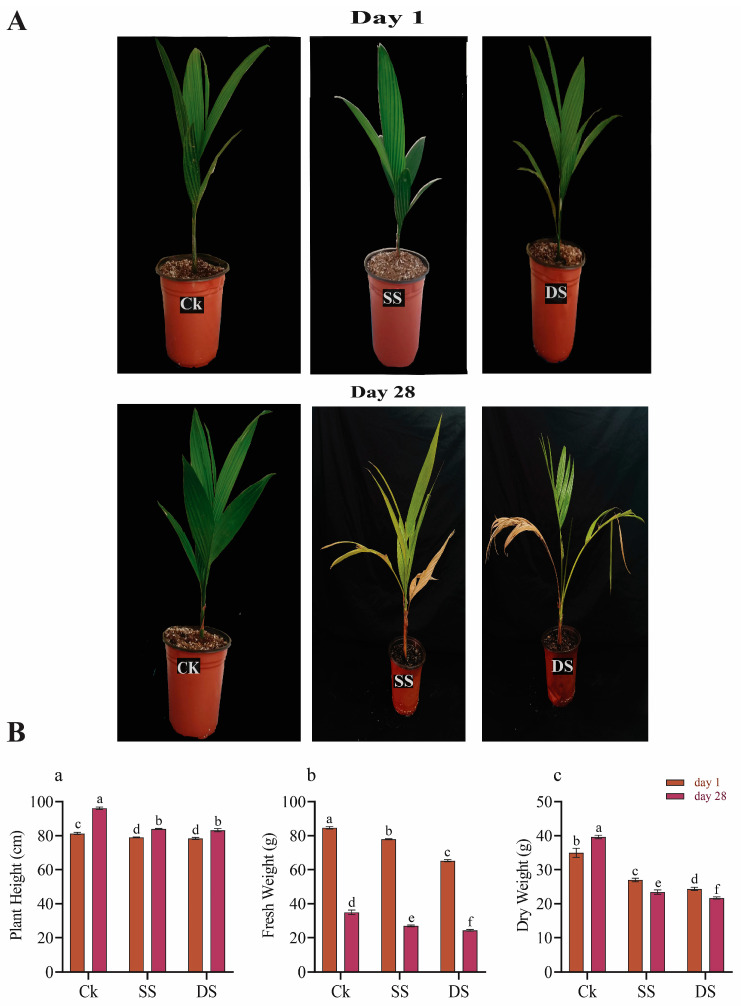
(**A**) The presentation of phenotypic changes observed on Day 1 and Day 28 of abiotic stress. (**B**) The physiological changes observed on Day 1 and Day 28 under abiotic stress (salt and drought): (a) plant height, (b) plant fresh weight, and (c) plant dry weight for CK, SS, and DS groups. Different lowercase letters represent the significant statistical level *p* < 0.05.

To analyze the phenotypic changes of plants under control (CK), salt stress (SS), and drought stress (DS), observations were made on Day 1 and Day 28. The results showed that all plants exhibited normal growth on Day 1 of exposure to abiotic stress. However, by Day 28, plants in the CK group displayed robust growth, while plants subjected to SS and DS exhibited visible signs of stress, including yellowing and leaf dryness (Figure 9A).

The plant height in the CK group was observed to be greater at 28 days compared to Day 1 and higher than in the SS and DS groups at 28 days (Figure 9B). However, the fresh weight of plants in the CK, SS, and DS groups was higher on Day 1 than at 28 days, with the CK plants showing the greatest fresh weight overall, compared to SS and DS. Additionally, the dry weight of CK plants at 28 days was significantly higher than that of CK, SS, and DS plants measured on both Day 1 and at 28 days.

### 2.10. Effects of Different Treatments on AcbHLH Expression

**Figure 10:**
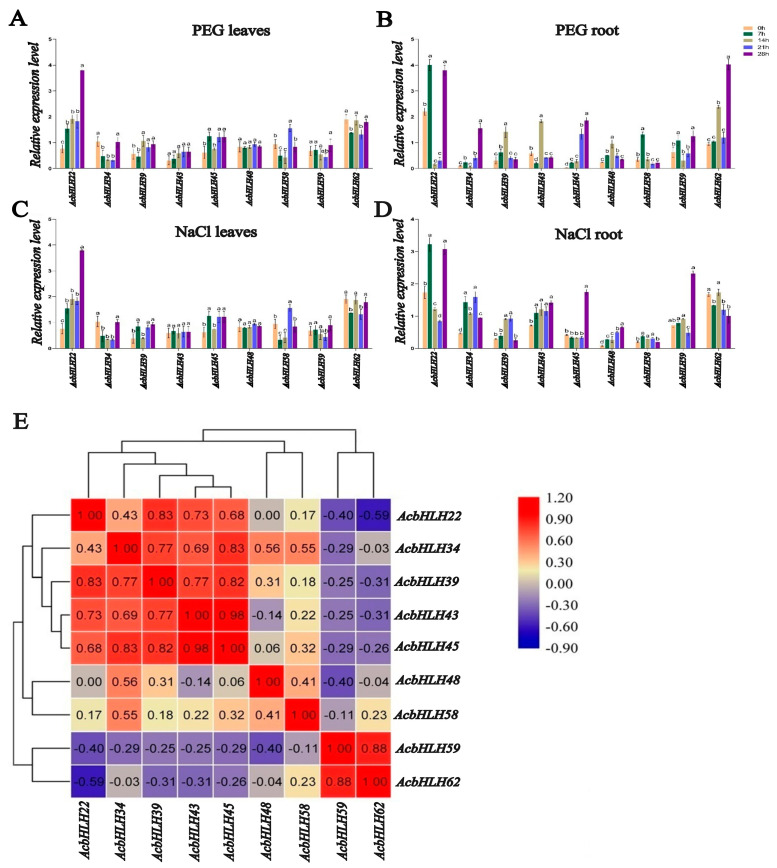
(**A**–**D**) The effects of abiotic stress (NaCl and PEG) detected by qRT-PCR on the expression of nine *AcbHLH* genes in roots and leaves of young *A. catechu* seedlings (0, 7, 14, 21, and 28 h) (*p* < 0.05). (**E**) Heatmap of nine *AcbHLH* genes. Different letters indicate statistically significant group differences (*p* < 0.05, LSD).

To investigate the role of *AcbHLH* genes in response to abiotic stress, we evaluated the expression levels of nine selected *AcbHLH* genes (*AcbHLH22*, *AcbHLH34*, *AcbHLH39*, *AcbHLH43*, *AcbHLH45*, *AcbHLH48*, *AcbHLH58*, *AcbHLH59*, and *AcbHLH62*) under SS and DS in both roots and leaves, using quantitative reverse-transcription PCR (qRT-PCR) (Figure 10A–D). The results revealed a variety of gene expression responses to both stress treatments, with distinct activation or inhibition patterns observed at different time points. Under DS, the expression of *AcbHLH22* was significantly upregulated in leaves at 28 days, while *AcbHLH39* showed a peak at 14 days, and AcbHLH45 was upregulated at 7, 21, and 28 days. Additionally, *AcbHLH58* showed significant upregulation in leaves at 21 days. In the roots, the expression of all nine genes was significantly upregulated at various time points under DS.

Under SS, specific genes exhibited differential expression in both leaves and roots. *AcbHLH22* was significantly upregulated in leaves at 21 and 28 days, *AcbHLH39* at 28 days, *AcbHLH45* at 7, 21, and 28 days, and *AcbHLH58* at 21 days. In roots, *AcbHLH22* was significantly upregulated at 7 and 28 days, *AcbHLH34* at 7 and 28 days, and *AcbHLH39* at 14 and 21 days. Other genes, such as *AcbHLH43*, *AcbHLH45*, *AcbHLH28*, and *AcbHLH59*, were significantly upregulated at 28 days, while *AcbHLH58* showed significant upregulation at 7 days. These findings demonstrate the temporal and tissue-specific regulation of *AcbHLH* genes under salt- and drought-stress conditions, shedding light on their potential roles in stress adaptation.

## 3. Discussion

Transcription factors control several biological processes by controlling the expression of certain genes, such as growth, development, and stress responses in plants [36]. The latest investigations show that the *A. catechu* plant has 31,571 protein-coding genes on 16 chromosomes [37]. Among these genes, a significant portion are classified as transcription factors [31]. Only a small number of the transcription factor families, including the DOF [38], WRKY, and MYB families (paper under review), have been systematically investigated in *A. catechu*. The bHLH TFs family, the second-largest among eukaryotes, is recognized for its evolutionary and functional diversity among plant species. However, the *bHLH* genes have not yet been characterized in *A. catechu.* In this study, we identified 76 *AcbHLH* genes that display a range of physicochemical characteristics, including differences in coding sequence length, chromosome number, molecular weight, protein length, GRAVY, isoelectric point (pI), number of exons/introns, subcellular localization, instability and aliphatic index (Table S1B). We used GRAVY values to indicate the average hydropathy of each peptide or protein. Our hydrophobicity research found that the *bHLH* gene family has negative GRAVY values, implying that they are hydrophilic, which aligns with those previously investigated [39].

Based on phylogenetic analysis, we categorized 76 *AcbHLH* genes and 162 *AtbHLH* proteins into 24 sub-families, emphasizing their evolutionary links and functional variety (Figure 1 and Table S1A). Our findings highlight evolutionary connections between *A. thaliana bHLH* genes (*AtbHLH*) and their counterparts in *A. catechu* (*AcbHLH*), with specific groupings showing close gene pair interactions, such as *AtbHLH47*-*AcbHLH31*, *AtbHLH21*-*AcbHLH77*, *AtbHLH13*-*AcbHLH83*, and *AtbHLH78*-*AcbHLH5*, among others. These findings, consistent with previous research into tomato, maize, and barley [40,41], indicated that *QbHLH* transcription factors have preserved evolutionary and functional properties across plant species, highlighting their extensive role in plant adaptation and development. Sequence alignment results of *AcbHLH* genes in *A. catechu* revealed consistent patterns of amino acid motifs (S, R, K, P, L, A, G, E, Y, I, V, H, M, Q, N, and D), as shown in Figure 2. As previously observed, these motifs were shared by *bHLH* proteins from various plant species, such as *A. thaliana* [42,43], *F. tataricum* [44], and *S. lycopersicum* [45].

The phylogeny and gene structure analysis of *AcbHLH* genes, as shown in Figure 3A,B and Table S1A, show that genes from the same subfamily have similar intron–exon structures. This indicated that changes in exon–intron distribution influence the evolution and functional diversity of *AcbHLH* proteins. Notably, the study discovered that the intron–exon number of *AcbHLH* genes varies from 1 to 13 (Figure 3B), indicating evolutionary changes such as insertions, deletions, and exon gains/losses. Furthermore, the relationship between intron number and gene expression in plants suggests that a more compact gene structure may allow for rapid gene expression in response to environmental stimuli [46], providing insight into the evolutionary adaptations of these genes in various biological processes. The conserved motif of AcbHLH proteins contained bHLH domains 1 and 2, as shown in Figure 3C. Additionally, the composition of other motifs was distinctive, and they were conserved across subgroups. For instance, motif 4 is exclusively present at the end of subgroups 3, 5, and 6. Other plants have been observed to exhibit similar phenomena [26,47].

Cis-acting elements, which are substances that bind to trans-acting factors, play a crucial role in regulating the activity of target genes [48]. These elements played an important role in molecular controlling genes, especially in response to stress expression [49]. Promoter analysis of the 2000 bp upstream regions of *AcbHLH* genes revealed 76 cis-elements classified into four main categories, as shown in Figure 4B: (I) growth-related (AAGAA, as-1), (II) phytohormone-responsive (ABRE, AuxRR-core), (III) light-responsive (G-box, TCT-motif), and (IV) stress-responsive (DRE, Myb, Myc). These elements are linked to responses to abiotic stresses like drought, temperature extremes, and wounding, as well as hormones like ABA and SA, implying that *AcbHLH* genes play an important role in regulating stress responses via hormone signaling (Figure 4A); this is as described by previous research, in which the *bHLH* gene, specifically *AtbHLH122* in *A. thaliana*, plays a vital role in ABA signaling pathways, increasing drought tolerance by regulating ABA metabolism and catabolism.

Research has revealed that gene duplication events are essential in several gene families’ rapid growth and expansion [50]. In the genome of *A. catechu*, four tandem duplications and twenty-three segmental duplication events were found in the *AcbHLH* family (Figure 5 and Table S2). These results provide strong evidence that segmental and tandem duplication had a role in the growth and divergence of the *AcbHLH* gene. Previous research showed segmental duplication has a more substantial role in genome evolution than tandem duplication [51]. We used Dup-206 Gene Finder to analyze gene duplication events in the *AcbHLH* gene family, identifying 23 duplicated genes in DSD, 11 in WGD, and 4 in PD (Figure 6 and Table S3D). Ka/Ks ratios for duplicated genes were all less than 1, indicating strong purifying selection (Figure 6B–F), aligning with previous study [52].

Gene functions within a gene family are frequently conserved across plant species, although this is not always possible. As a result, it is important to precisely determine the orthologs across plant species using synteny analysis. The result showed that *AcbHLH*63 in the Areca genome exhibited significant synteny with *A. thaliana*, *V. vinifera*, *S. lycopersicum*, *B. distachyon*, *O. sativa*, and *Z. mays* (Figure 7 and Table S4D). These results indicate that these genes may have played a crucial role in the evolutionary history of *A. catechu*, potentially undergoing loss or significant differentiation.

Furthermore, to investigate the role of *AcbHLH* genes in response to abiotic stress, we evaluated the expression levels of nine selected *AcbHLH* genes (*AcbHLH22*, *AcbHLH34*, *AcbHLH39*, *AcbHLH43*, *AcbHLH45*, *AcbHLH48*, *AcbHLH58*, *AcbHLH59* and *AcbHLH62*) under NaCl and PEG stresses in both roots and leaves, using quantitative reverse-transcription PCR (qRT-PCR) (Figure 10A–D). Under PEG stress, *AcbHLH22*, *AcbHLH39*, *AcbHLH45*, and *AcbHLH58* exhibited distinct upregulation in leaves at specific time points, with *AcbHLH22* being most strongly expressed at 28 days, *AcbHLH39* peaking at 14 days, and *AcbHLH45* showing sustained expression at 7, 21, and 28 days. These results are consistent with previous findings in other species, where *bHLH* genes demonstrated differential expression patterns under abiotic stress [53]. For example, in *C. quinoa*, eleven *bHLH* genes were significantly upregulated in response to cold stress, while eight were downregulated, suggesting a complex regulation of *bHLH* genes under stress [7,25]. Similarly, in *S. bicolor*, a range of *bHLH* genes exhibited both up- and downregulation in response to various abiotic stresses, supporting the idea of *bHLH* genes as key regulators in stress responses [9,54]. Our findings shows that *AcbHLH* genes, particularly *AcbHLH22* and *AcbHLH39*, are strongly upregulated under DS, suggesting their role in drought tolerance mechanisms.

In the roots, the expression of all nine *AcbHLH* genes was upregulated under PEG stress at various time points, which aligns with the general response of *bHLH* genes to abiotic stresses in other plants [55]. These results imply that *AcbHLH* genes are involved in leaf stress responses and play a crucial role in root adaptation to osmotic stress [10,56]. Under SS, our study found that *AcbHLH22*, *AcbHLH39*, *AcbHLH45*, and *AcbHLH58* exhibited upregulation in leaves, with *AcbHLH22* showing significant expression at both 21 and 28 days. In roots, *AcbHLH22* was upregulated at 7 and 28 days, while *AcbHLH34* was upregulated at 7 and 28 days, and *AcbHLH39* showed peak expression at 14 and 21 days. These results support the fact that *bHLH* genes regulate tissue-specific stress responses, as seen in other species like *Z. mays*, where *bHLH* genes such as *ZmbHLH180* and *ZmbHLH23* exhibited synergistic expression patterns under stress [11,57].

Interestingly, *AcbHLH22* was significantly upregulated under both salt and drought stress in our study, which contrasts with findings in *A. thaliana*, where *bHLH* genes like *AtbHLH22* did not show similar upregulation under abiotic stress [12,58]. This discrepancy suggests that *AcbHLH22* might have a unique role in *A. catechu* that warrants further investigation, particularly in relation to its involvement in stress-induced stomatal regulation. Previous studies have linked bHLH transcription factors to regulating leaf stomatal development, which is crucial for maintaining water balance under stress [13,59]. This opens avenues for exploring how *AcbHLH22* may influence water use efficiency and osmotic regulation in response to environmental stresses. This suggests a potential synergistic interaction between the two genes, likely coordinating their physiological roles, particularly under abiotic stress and during vegetative development [14,60]. This study provides the comprehensive analysis of the bHLH transcription factor family in *A. catechu*, offering valuable insights into their evolutionary diversity and potential roles in abiotic stress responses. While the findings lay a strong foundation, addressing the outlined limitations and exploring the suggested future directions will further enhance our understanding of *AcbHLH* gene functions, enabling their application in crop improvement strategies for stress resilience.

## 4. Materials and Methods

### 4.1. Identification of AcbHLH Genes in A. catechu

The complete genome sequence of *A. catechu* L. was obtained from the National Center for Biotechnology Information (NCBI) using the following (ID: JAHSVC000000000; BioSample: SAMN19591864; Accession: PRJNA735650). The bHLH protein domain (Pfam ID: PF00010) was retrieved via the Pfam database. The conserved bHLH protein domain (Pfam ID: PF00010) was retrieved from the Pfam database. A two-step approach was employed to identify *AcbHLH* within *A. catechu*. The first approach, the protein sequences of *Arabidopsis* bHLH used as query sequences downloaded from TAIR (http://www.arabidopsis.org) (accessed on 15 March 2024), apply BLASTp with a score value of at least 100 and an E-value of less than or equal to 1 × 10^−10^. In the second approach, the *bHLH* candidates were initially identified within the *A. catechu* genome through a hidden Markov model (HMM) search, employing an e-value lower than 10^−5^, based on a previously used method [15]. Finally, unidentified conserved sequence motifs were manually removed from the dataset. To validate the presence of bHLH domains, HMMER R3.0 (http://plants.ensembl.org/hmmer/index.html) (accessed on 20 March 2024) [16] with default parameters and a 0.01 cutoff was used for protein homology, and confirmation was achieved using the SMART domain database (http://smart.embl-heidelberg.de/) (accessed on 24 March 2024).

### 4.2. Analysis of Physicochemical Properties of AcbHLH

The ExPasy ProtParam server (http://web.expasy.org/protparam/) (accessed on 30 March 2024) was utilized to characterize the fundamental characteristics of *AcbHLH* tri-helix proteins, sequence length, isoelectric point (pI), and molecular mass within the *AcbHLH* gene family. A protein subcellular localization prediction tool (PSORT) was used to determine the likely cellular location of the predicted proteins (https://www.genscript.com/psort.html) (accessed on 8 April 2024) [17].

### 4.3. The bHLH Gene Structure, Conserved Motif, and Cis-Acting Elements

The characterized *AcbHLH* proteins were aligned manually using ClustalW, and GeneDoc software version 2.7 was used to improve the alignment quality manually. An in-depth analysis of the intron–exon structure of *AcbHLH* genes in *A. catechu* was achieved by the Gene Structure Display Server (GSDS) (http://GSDS.cbi.pku.edu.cn/) (accessed on 14 April 2024) by leveraging the GFF3 annotation data. The online tool MEME was used to identify motifs of AcbHLH proteins (http://meme-suite.org/tools/meme) (accessed on 20 April 2024). Promoter sequences of *AcbHLH* genes were retrieved from the *A. catechu* genome (http://bioinformatics.psb.ugent.be/webtools/plantcare/html/) (accessed on 27 April 2024). Upstream regulatory sequences of these genes were identified as the 2000 base pairs preceding the ATG codon. The distribution of cis-elements within the promoter regions was analyzed using the PlantCARE database (http://bioinformatics.psb.ugent.be/webtools/plantcare/html/) (accessed on 8 May 2024).

### 4.4. Chromosomal Distribution and Gene Duplication

Chromosomal localization of *AcbHLH* was determined by mapping their physical location to the *A. catechu* genomic database and Circular Genome Data Visualization tool (Circos). The Multiple Collinearity Scan toolkit (MCScanX) was employed to analyze gene duplication events, using its parameters [18]. A dual synteny plotter was used to analyze gene homogeneity among *A. catechu* and *A. thaliana* (https://github.com/CJ-Chen/TBtools) (accessed on 12 May 2024). Nonsynonymous and synonymous substitution rates were calculated for each duplicated *bHLH* gene using the Ka/Ks Calculator 2.0 [19].

### 4.5. Evolutionary Relationships and Classification of AcbHLH Gene Family

All the recognized genes, *AcbHLH*, were classified based on *AtbHLH* categorization. The phylogenic tree was created utilizing the Neighbor Joining (NJ) method in MEGA-X software 11.0.10; using 1000 bootstrap replicates and the NJ method, the phylogenetic tree was created and visualized using iTOL [19]. For phylogenetic analysis, the full-length amino acid sequences of *A. thaliana bHLH* proteins were utilized, (https://itol.embl.de/) (accessed on 15 May 2024).

### 4.6. RNA-Seq Data Analysis

The expression profile study of *AcbHLH* genes was conducted using Illumina Hisequence 4000 RNA-sequence data submitted to the NCBI database (accession number: PRJNA767949). Genes exhibiting a log2FC fold change greater than 1, and a false discovery rate (FDR) of 0.05 with 3 biological replicates per treatment and differential expression were identified using a significant threshold of *p*-value < 0.05. The expression pattern of *AcbHLH* genes was plotted using TBtools. Gene expression studies were conducted using the BMK Cloud platform (https://www.biocloud.net/) (accessed on 20 May 2024).

### 4.7. Plant Materials, Growth Conditions, and Abiotic Stress in A. catechu

The research utilized seedlings of *A.catechu* (Reyan No. 1) from the Coconut Research Institute of the Chinese Academy of Tropical Agricultural Sciences, located in Wenchang, Hainan province, China. Seedlings were grown in pots (12 cm × 12 cm) at 14/10 h day/night at 28/25 °C. The plants were treated, including drought (DS) (25% PEG6000) and salt (SS) (5% NaCl) [7]. Leaf and root samples were collected at 1, 7, 14, 21, and 28 days for each treatment and preserved at −80 °C for s subsequent analysis.

### 4.8. Phentypic and Physiological Analysis

Phenotypic changes were observed on Day 1 and Day 28 in the CK, SS, and DS groups. Plant height was measured on both days, using a measuring tape. Fresh weight was determined using a weighing balance for samples collected on Day 1 and Day 28. For dry weight analysis, plant samples from the control and treatment groups were dried in an oven (DGG-9053A, Shanghai Enxin Instrument Co., Ltd., Shanghai, China) at 65 °C for 72 h and then weighed using a balance [61].

### 4.9. Total RNA Extraction, cDNA and qRT-PCR

Total RNA extraction from plant samples was performed using a specialized extraction kit (TIANGEN, Beijing, China). RNA concentration and purity were checked using NanoDrop 2000 (KAIAO, Beijing, China). First-strand cDNA synthesis was performed using the TIANScript RT Kit (TIANGEN, Beijing, China). Quantitative real-time PCR (qRT-PCR) was used to analyze gene expression using Vazyme Master Mix (Vazyme, Nanjing, China). PCR conditions were set according to the company manual. Primers were designed via Primer 6.0 software, and *Actin* was used as an internal control for gene expression analysis (Table S6). The 2^−ΔΔCT^ method was utilized to analyze qRT-PCR data and calculate relative gene expression levels.

### 4.10. Statistical Analysis

Analysis of variance (ANOVA) was analyzed by Statistics 8.1 software, followed by the Tukey LSD test, and compared with the least significant difference (LSD) (*p* ≥ 0.05). The histogram was drawn using Origin 8.0 software (OriginLab, Northampton, MA, USA).

## 5. Conclusions

This research represents the first comprehensive and systematic evaluation of the *AcbHLH* gene family within the *A. catechu* genome, encompassing a wide range of analytical approaches and verifications. A total of 76 *AcbHLH* gene–proteins were identified and categorized into 24 distinct subgroups based on their protein domain profiles and gene structure, which carry the categorization accuracy based on phylogenic analysis and are irregularly distributed on 16 chromosomes (Chr00). The distribution of *AcbHLH* genes across the chromosomes was not evenly distributed. Some *AcbHLH* genes are involved in gene replication events, and fragment repeat contributes more favorably than tandem duplication. A significant homologous phenomenon revealed the homology between one *AcbHLH* gene and multiple *bHLH* genes in *A. Catechu*. The *AcbHLH* genes from *A. catechu* are the most closely related genes among the six representative plant species, as determined by sequence comparison. Finally, qRT-PCR analysis revealed that nine *AcbHLH* genes were differentially expressed in response to abiotic stress conditions. The expression mechanism of these genes was investigated throughout the developmental stages, and *AcbHLH22* and *AcbHLH144* demonstrated a notable impact on abiotic stress resistance. It is speculated that *AcbHLH22* and *AcbHLH62* are essential components of the *A. catechu* life cycle, contributing to its viability and development. In conclusion, this research offers valuable insights into the biological functions and development of *AcbHLH* in *A. catechu* and will facilitate future research on the functions of *AcbHLHs*.

## Data Availability

The data generated and analyzed in this study are available on the request.

## Supplementary Materials

The following supporting information can be downloaded at: https://www.mdpi.com/article/10.3390/ijms252312936/s1.
